# Human Bone Marrow-Derived Mesenchymal Cell Reactions to 316L Stainless Steel: An in Vitro Study on Cell Viability and Interleukin-6 Expression

**DOI:** 10.15171/apb.2017.040

**Published:** 2017-06-30

**Authors:** Iwan Budiwan Anwar, Asep Santoso, Eko Saputra, Rifky Ismail, J. Jamari, Emile Van der Heide

**Affiliations:** ^1^Laboratory for Surface Technology and Tribology, Faculty of Engineering Technology, University of Twente Drienerloolaan 5, Postbox 217, 7500 AE, Enschede, The Netherlands.; ^2^Orthopaedic and Traumatology Department, Prof. Dr. R. Soeharso Orthopaedic Hospital, Jl. A. Yani Pabelan, Surakarta 57162, Indonesia.; ^3^Laboratory for Engineering Design and Tribology, Department of Mechanical Engineering, Diponegoro University, Jl. Prof. Soedharto, Tembalang, Semarang 50275, Indonesia .

**Keywords:** 316L stainless steel, Human bone marrow-derived mesenchymal cells, Cell viability, Interleukin-6

## Abstract

***Purpose:*** Human bone marrow-derived mesenchymal cell (hBMC) reactions to 316L stainless steel (316L-SS) have never been evaluated. The objective of this study was to assess cell viability and interleukin-6 expression of hBMC cultures upon treatment with a 316L-SS implant.

***Methods:*** A cytotoxicity analysis was conducted with a 3-(4,5-dimethylthiazol 2-yl)-2,5-diphenyltetrazolium (MTT) assay after a period of 24, 48 and 72 hours of incubation. Expression of interleukin-6 was measured using enzyme-linked immunosorbent assay (ELISA).

***Results:*** Cell viability measurement was performed via IC50 formula. All treatment group showed a > 50 % cell viability with a range of 56,5 - 96,9 % at 24 hours, 51,8-77,3% at 48 hours and 70,1- 120 % at 72 hours. Interleukin-6 expression was downregulated subsequent to treatment with 316L-SS compared to the control group.

***Conclusion:*** We found that 316L-SS did not exhibit toxicity towards hBMC culture.

## Introduction

Presently, 316L stainless steel (316L-SS) implants are still widely utilized in the field of medical practice, especially orthopedic surgery. It has excellent mechanical properties, corrosion resistance and is cost effective.^1^ It is mandatory to evaluate biomedical safety before using such implants in humans.^2^ As one of the dominant cellular components of medullary bone marrow, mesenchymal cells have important roles in several human tissue regeneration processes. They also possess the capability of producing several cytokines (include interleukin-6/IL-6) after contact with foreign material.^3,4^ Human bone marrow mesenchymal cells (hBMC) reactions to 316L-SS have never been evaluated. Therefore, herein, we conducted an *in vitro* cytotoxicity study of 316L-SS on hBMC along with evaluating the expression of IL-6.

## Materials and Methods

### Human bone marrow-derived mesenchymal cell isolation and culture

Bone marrow samples were obtained from a patient experiencing total hip arthroplasty surgery with written informed consent and ethical approval from the Ethics Commission of Prof. Dr. R. Soeharso Orthopedic Hospital, Solo, Indonesia. During surgery, an approximately 10 ml of bone marrow suspension was harvested from the intramedullary canal of the femur. It was captured in a 20 ml tube (Falcon, BD Bioscience) containing the same volume of heparinized (10 U/mL) phosphate – buffered saline (PBS) to prevent clotting. The mixture of bone marrow and heparinized PBS was kept at 4°C prior to further processing in the laboratory. hBMC culturing was conducted in the Laboratory of Cell Culture at the Department of Physiology, Gadjah Mada University School of Medicine, Yogyakarta, Indonesia.

Bone marrow was loaded into a centrifuge tube and centrifuged at 2500 rpm for 10 minutes at room temperature. The top layer of mononuclear cells was collected and washed with PBS twice and Dulbeco’s Modified Eagle Media (DMEM) once. The isolated cells were suspended in DMEM, supplemented with 15% PBS, 1% antibiotic – mycotic (Sigma – Aldrich, Co, USA) and seeded into 25 cm^2^ flasks. The cells were incubated at 37°C, 5% CO2. After three days, non-adherent cells were removed by washing twice with PBS and the new medium was applied. The cell density and morphology was monitored under an inverted microscope. When the primary cultured cells reached at least 80% confluence, they were harvested using 0.25% trypsin (Sigma – Aldrich, Co, USA) and subcultured. The third passage of cells was used for subsequent studies.

### Immunohistochemistry 

**Figure 1:**
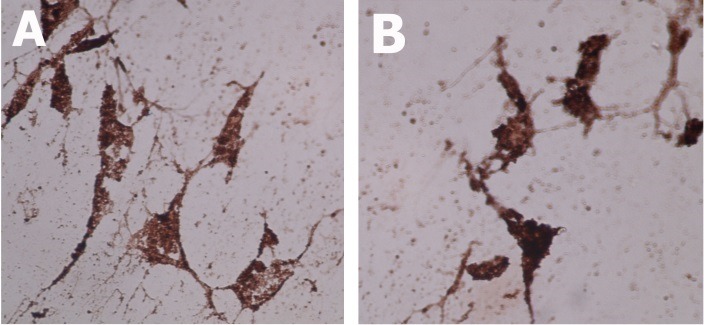
hBMC showed positive immunohistochemistry staining of both CD-44 (A) and CD-19 (B) appeared in dark brown color (x40).

Confirmation of mesenchymal cell markers was carried out with immunohistochemistry (IHC) staining. Cells were ﬁxed with 4% paraformaldehyde, blocked to prevent non-speciﬁc antibody binding and incubated with primary antibodies at 4°C overnight. Following PBS washing, anti-CD-44 and anti-CD-19 were used for human BMSC (®Bioss). All conditions were maintained in negative controls excepting that the primary antibodies were eliminated. Dishes were examined under a ﬂuorescence microscope (Nikon Eclipse E400). CD-44 was employed as a positive marker of mesenchymal lineage cells and CD-19 as a positive marker of hematopoietic lineage cells (Figure 1A, B).

### Cytotoxicity study of 316L-SS on hBMC

The resultant hBMC from primary culture was re-cultured on 96-well microplates, one 96-well microplate being prepared for each incubation period: 24, 48 and 72 hours. The 316L-SS implants were placed in the wells before seeding the stem cells. The implants consisted of two types - polished and non-polished. Implant polishing was with toluene liquid. The implants also had two different sizes - 316L-SS with a diameter of 2 mm (size A) and 316L-SS with a diameter of 4 mm (size B). Both had 1 mm thickness. Each type and size of implant was duplicated into three (N = 3 per group). 1x10^4^ cells were seeded in each well. After the seeding process, the plates were incubated at 37°C, 5% CO_2_ for 24, 48 and 72 hours. We evaluated the cells at the end of each incubation period under an inverted microscope. Culture medium was also collected for IL-6 expression measurement with an immunoassay kit (Komabiotech, ELISA).

Each well of the 96-well microplates evaluated for cytotoxicity had 50 μl solution containing 5 mg/ml of 3-(4,5-dimethylthiazol 2-yl)-2,5-diphenyltetrazolium (MTT) reagent in PBS added, and then the microplates were reincubated for 4 hours in CO_2_ 5% at 37°C. Colorimetric assay with MTT was applied to assess viability of the cells after 24, 48 and 72 hours of treatment with 316L-SS implants. Next, to each well was added with 50 μl of dimethyl sulfoxide (DMSO), and the microplate was then reincubated for 5 minutes at 37°C. The optical density of each well was measured using an ELISA reader at 620 nm wavelength.

Optical density data were tabulated and analyzed using the *t*-test (SPSS v17, Chicago, USA) at a significance of 95%. Percentage of viable hBMC were calculated using the IC_50_ formula:^5^

$$\text{Percentage of viable cells}=\frac{\text{Optical density of experiment group }-\text{ Optical density of control media}}{\text{Optical density of control cells }-\text{ Optical density of control media}}\times 100\text{\%}$$

## Results and Discussion

### Microscopic evaluation and cytotoxicity study

**Figure 2:**
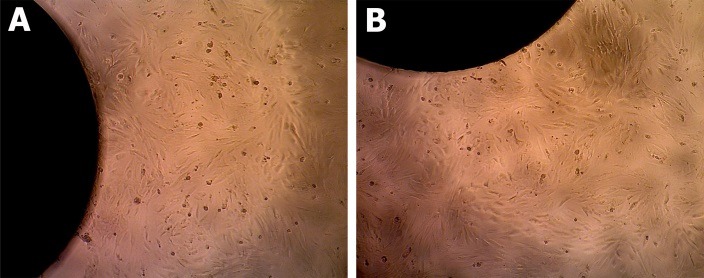
Microscopic evaluation of hBMC around 316L-SS implants. Cells attached to both polished (A) and non-polished implant (B) after 24 hours incubation (x10).

The visual microscopic evaluation was performed on all study groups before carrying out the MTT assay using an inverted microscope. We found that hBMC attached to the implant in all experiment groups (Figure 2A, B). According to the MTT assay results, the control group had greater optical density compared to the treatment group for all (24, 48 and 72 hours) incubation periods. Statistical analysis revealed that the 24-hour incubation period for all implants did not cause a significant difference in cell optical density compared to controls. Yet, a significant difference (*p*<0.05) relative to the control group was observed at 48 hours for non-polished B and at 72 hours for polished A and B (Table 1).

Statistical analysis was also performed to evaluate the cell optical density differences between the period of incubation for each implant in the control and treatment group. It was showed that neither in the control or treatment groups was a significant difference (*p*<0.05) observed between the 24-hour and 72-hour groups, and this was also true when comparing the 48-hour and 72-hour groups. No significant differences between the 24-hour and 48-hour groups of controls with all experimental groups were found. Cell viability measurement was performed with the IC_50_ formula. At 24, 48 and 72 hours, all treatment group exhibited a > 50 % cell viability (Table 2).

**Table 1:** Mean and standard deviation of cells optical density of all study groups.

Groups	N	Mean ± standard deviation		
		24 hours	48 hours	72 hours
Polished-A	3	0.363 ± 0.024	0.340± 0.032	0.437± 0.015*
Polished-B	3	0.339± 0.008	0.328± 0.011	0.419± 0.013**
Nonpolished-A	3	0.375± 0.013	0.353± 0.025	0.481± 0.045
Nonpolished-B	3	0.335± 0.008	0.310±0.014***	0.479±0.017
Control Cells	3	0.378±0.025	0.384±0.021	0.456±0.013
Control Media	3	0.279±0	0.247±0.004	0.332±0

Comparison to the control group at same period of incubation: * *p* = 0.03, **p = 0.00, *** *p* = 0.00.

**Table 2:** Results on viability of mesenchymal cells after contact to 316L-SS after each incubation period.

Cells Viability	24 hours	48 hours	72 hours
Polished A	84.8%	67.8%	84.6%
Polished B	61.6%	59.1%	70.1%
Non-Polished A	96.9%	77.3%	120%
Non-Polished B	56.5%	51.8%	118%

Several biocompatibility studies of 316L-SS with various human cells have been previously conducted. In one study by Li et al^6^ the biological behavior of human umbilical artery smooth muscle cells (HUASMC) was evaluated in cultures containing high nitrogen nickel-free (HNNF) stainless steel and 316L-SS material. They determined that HNNF stainless steel activated more cellular apoptosis, and also reduced cell proliferation in comparison to 316L-SS. Overall, 316L-SS appeared to be more biocompatible versus HNNF stainless steel.

In another study by Martinesi et al^7^ it was reported there was biocompatibility between surface-treated 316L-SS and human cell cultures containing human umbilical vein endothelial cells (HUVEC) and human peripheral blood mononuclear cells (PBMC). HUVEC proliferation and apoptosis decreased and increased, respectively, in the presence of the nitrided post-oxidized 316L-SS after 72 hours of contact. Meanwhile, human PBMC decreased in terms of proliferation with a concomitant increase in apoptosis in the presence of the untreated samples and those that were nitrided post-oxidized after 48 hours of incubation.

In our study, we found a lower cell viability of hBMC after contact with polished 316L-SS compared to non-polished 316L-SS of the smaller implant group for all incubation period lengths. Taking into account the results of the cell optical density measurements, we established there was no significant difference in optical density between the 24-hour and 48-hour groups for all types of implants. This indicated that there may be changes in cell viability that predominantly take place during the period between 48 to 72 hours. Despite the decrease in cell viability with several of the treatment groups, we still obtained more than 50% viable cells based on IC_50_ formula computation. Our study and those completed earlier suggest that each type of human cell could react differently to 316L-SS.

### Expression of IL-6

**Figure 3:**
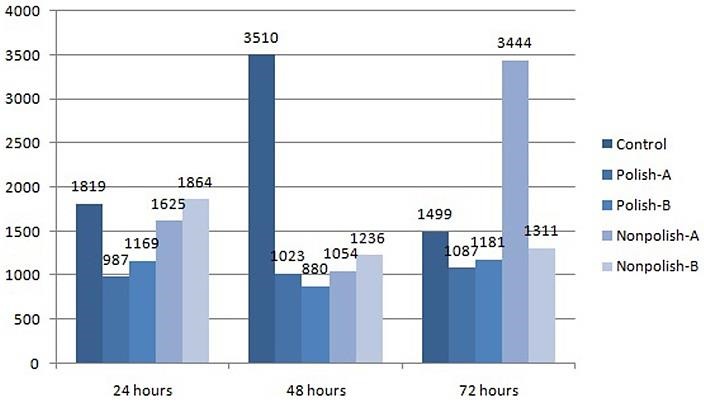
Results of interleukin-6 expression of hBMC on each incubation period (*pg/ml*).

There was a lower expression of IL-6 in hBMC compared to controls for all polished implants with all incubation periods. The situation was similar for the non-polished implant group, except with 72 hours of incubation and the small non-polished implant. In addition, the polished implant induced lower expression of IL-6 compared to non-polished implants for all treatment groups (Figure 3).

Martinesi et al^7^ described a remarkable increase in IL-6 expression in human PBMC after 316L-SS treatment. However, they saw that 316L-SS only causes a slight difference in IL-6 expression with HUVEC culturing. Here, we found a lower expression of IL-6 by hBMC that received treatment by polished implants compared to non-polished implants. This finding may indicate that a polished implant has a more inert surface and brings about less of a cell reaction compared to non-polished implants.

## Conclusion

In this study, we found 316L-SS did not exhibit any toxic effects toward human bone marrow-derived mesenchymal cells cultures. Polishing 316L-SS implants appeared to reduce cellular reactions.

## Acknowledgments

This study was funded by PUSNAS research grant of the Ministry of Research, Technology and Higher Education of Indonesia, Based on the Agreement Letter: 022/SP2H/LT/DRPM/II/2016 on I 7 February 2016.

## Ethical Issues

Not applicable.

## Conflict of Interest

The authors declare no conflict of interest.
